# Supplementary material for: Self-Reporting of Post-Vaccination Symptoms in the COVID-19 Vaccination Process for Teachers in a North Region of Poland
Source: Vaccines (Basel). 2025 Oct 14;13(10):1054. doi: 10.3390/vaccines13101054 (PMC12567939; doi:10.3390/vaccines13101054)
Supplement: Supplementary file 1 [file vaccines-13-01054-s001.zip › vaccines-3874470-supplementary.pdf]

Table S1. Intensity of late post-vaccination self-reported symptoms compared to symptoms of recent infection.

Feature	much weaker		weaker		hard to say		stronger		much stronger		log [BF]
	%	95% BCI	%	95% BCI	%	95% BCI	%	95% BCI	%	95% BCI	
**Musculoskeletal pains**											
**Female**	3.85	3.11-4.78	10.25	8.92-11.77	7.37	6.27-8.67	44.6	42.4-46.85	33.87	31.17-36.37	
**Male**	5.7	4.24-7.45	14.15	11.43-17.22	9.33	7.72-11.17	45.29	43.01-47.65	25.23	21.08-30.25	**-3.7**
**I don't want to answer that question**	3.39	2.08-5.46	9.15	6.08-13.41	6.75	4.64-9.11	43.67	38.72-46.84	36.8	26.96-48.13	
**20-30 years**	2.84	2.01-4.04	7.87	5.96-10.35	5.95	4.58-7.56	42.01	38.46-45.33	41.22	34.35-48.25	
**31-40 years**	3.22	2.45-4.21	8.78	7.19-10.62	6.51	5.32-7.88	43.28	40.79-45.84	38.08	33.61-42.74	
**41-50 years**	3.91	2.99-5.07	10.4	8.61-12.45	7.44	6.14-8.95	44.68	42.34-47.04	33.39	28.27-37.91	**8.63**
**51-60 years**	5.1	3.91-6.53	12.93	10.85-15.36	8.78	7.35-10.46	45.42	43.16-47.04	27.54	23.85-31.67	
**61-70 years**	7.08	5.02-9.85	16.77	12.96-20.95	10.4	8.41-12.46	44.34	41.22-46.98	21.1	16.34-26.84	
**Headaches**											
**Female**	3.43	2.67-4.33	10.47	9.08-11.99	9.46	8.23-10.81	39.97	37.81-42.24	36.59	34.01-39.25	
**Male**	6.61	4.93-8.84	17.65	14.63-21.25	13.41	11.42-15.68	39.61	36.92-42.18	22.43	18.49-26.88	**6.19**
**I don't want to answer that question**	4.06	2.42-6.89	11.98	7.85-17.87	10.47	7.45-13.7	40.23	37.09-42.71	32.7	22.89-44.39	
**20-30 years**	4.33	2.98-6.12	12.72	9.67-16.32	10.88	8.74-13.24	40.58	38.43-42.94	31.2	25.01-37.97	
**31-40 years**	3.55	2.66-4.65	10.73	8.73-13.01	9.66	8.06-11.54	40.08	37.87-42.49	35.86	30.95-40.45	
**41-50 years**	3.64	2.77-4.75	10.95	9.05-13.13	9.81	8.19-11.59	40.22	37.98-42.62	35.3	30.83-39.88	**1.97**
**51-60 years**	4.64	3.51-6.05	13.4	11.13-16.09	11.33	9.58-13.21	40.77	38.68-43.02	29.66	25.51-34.05	
**61-70 years**	7.33	5.18-10.45	18.9	15.23-23.7	13.92	11.66-16.3	38.89	35.42-41.7	20.71	15.92-26.92	
**Feeling tired/weak/broken**											
**Female**	2.38	1.85-3.02	5.93	4.99-6.93	5.19	4.33-6.09	42.73	40.66-44.96	43.77	41.16-46.25	
**Male**	3.56	2.59-4.85	8.48	6.62-10.58	7.01	5.63-8.63	46.77	43.93-49.52	34.1	29.04-39.35	**-3.3**
**I don't want to answer that question**	2.91	1.79-4.73	7.06	4.58-10.47	6.00	4.22-8.49	45.01	39.00-48.86	38.81	28.67-49.92	

**20-30 years**	1.94	1.34-2.82	4.92	3.66-6.47	4.4	3.35-5.72	39.85	35.14-43.98	48.82	41.93-55.95	
**31-40 years**	2.23	1.65-3.01	5.57	4.44-6.91	4.91	3.96-6.08	41.88	38.67-44.81	45.29	40.71-50.05	
**41-50 years**	2.73	2.03-3.63	6.7	5.39-8.25	5.75	4.72-7.02	44.42	41.55-47.08	40.3	36.04-44.94	**10.3**
**51-60 years**	3.54	2.65-4.69	8.45	6.91-10.32	7.01	5.81-8.42	46.87	44.38-49.17	33.91	30.08-38.33	
**61-70 years**	4.9	3.46-6.91	11.13	8.66-14.13	8.68	6.89-10.83	48.06	45.7-50.3	26.88	21.78-32.82	
**Shivers**											
**Female**	2.71	2.05-3.55	7.46	6.21-8.75	6.63	5.55-7.78	35.68	33.56-37.92	47.47	44.5-50.34	
**Male**	3.4	2.4-4.76	9.06	7.11-11.38	7.78	6.21-9.57	37.83	35.05-40.53	41.74	36.27-47.97	**-7.1**
**I don't want to answer that question**	2.17	1.22-3.71	6.06	3.7-9.74	5.54	3.5-8.34	32.76	25.27-38.75	53.34	40.59-65.85	
**20-30 years**	2.14	1.44-3.14	6.00	4.39-8.13	5.51	4.15-7.12	32,82	28.48-36.75	53.31	46.00-60.60	
**31-40 years**	2.29	1.65-3.13	6.42	5.1-8.08	5.82	4.66-7.15	33,65	30.49-36.69	51.7	46.54-56.90	
**41-50 years**	2.58	1.88-3.47	7.12	5.65-9.02	6.38	5.12-7.86	35,12	32.11-38.00	48.71	43.53-53.82	-2.9
**51-60 years**	3.04	2.18-4.13	8.22	6.62-10.31	7.19	5.82-8.82	36,9	34.16-39.59	44.57	39.54-49.62	
**61-70 years**	3.74	2.52-5.49	9.88	7.21-13.24	8.3	6.4-10.83	38.53	35.22-41.37	39.2	31.69-47.14	
**Concentration difficulties**											
**Female**	4.55	3.64-5.61	9.78	8.33-11.24	29.39	27.31-31.8	34.49	32.26-36.77	21.69	19.58-23.96	
**Male**	4.85	3.57-6.51	10.3	8.16-12.89	30.23	26.87-33.52	33.92	31.01-36.64	20.52	16.69-24.98	**-9**
**I don't want to answer that question**	4.8	2.8-7.86	10.23	6.4-15.21	30.05	23.15-35.56	33.93	28.49-37.66	20.8	13.58-30.78	
**20-30 years**	3.32	2.29-4.7	7,44	5.53-9.67	25.19	20.87-29.29	36.16	33.63-38.62	27.75	22.24-34.39	
**31-40 years**	3.54	2.64-4.69	7,78	6.31-9.68	26.02	22.83-29.17	36.02	33.66-38.36	26.5	22.47-30.87	
**41-50 years**	4.21	3.19-5.56	9,14	7.44-11.3	28.44	25.31-31.44	35.06	32.55-37.46	23.04	19.51-27.16	**9.35**
**51-60 years**	5.64	4.3-7.29	11,66	9.49-14.2	31.97	28.94-35.01	32.5	29.67-35.13	18.15	15.07-21.67	
**61-70 years**	8.34	5.87-11.8	15,77	12.25-19.86	35.41	32.3-38.39	27.55	22.93-31.89	12.72	9.37-16.65	
**Feeling irritable**											
**Female**	5.71	4.60-7.00	12.19	10.41-13.91	36.00	33.71-38.37	29.10	26.97-31.49	16.89	15.05-18.93	**-8.5**
**Male**	6.51	4.85-8.64	13.52	10.73-16.54	37.26	34.12-40.18	27.53	24.18-30.96	14.94	11.92-18.62	

**I don't want to answer that question**	5.77	3.45-9.46	12.26	8.12-17.64	36.13	29.59-40.39	28.98	22.98-34.00	16.62	11.04-25.16	
**20-30 years**	5.23	3.72-7.42	11.42	8.55-14.38	34.97	30.81-38.91	30.05	26.31-33.54	18.20	14.00-23.54	
**31-40 years**	4.70	3.64-6.06	10.38	8.43-12.55	33.72	30.64-36.84	31.17	28.41-33.90	19.94	16.64-23.58	
**41-50 years**	5.03	3.91-6.39	10.95	8.94-13.28	34.55	31.55-37.52	30.51	27.74-33.34	18.90	15.70-22.52	**0.68**
**51-60 years**	6.36	4.97-8.12	13.29	10.89-15.86	37.13	34.29-39.88	27.81	24.94-30.81	15.29	12.53-18.42	
**61-70 years**	9.52	6.78-13.33	18.00	13.87-22.14	39.38	36.69-41.84	22.45	18.12-27.04	10.41	7.60-14.41	
